# Mini Review: Correlations of Cognitive Domains With Cerebrospinal Fluid α-Synuclein Levels in Patients With Parkinson's Disease

**DOI:** 10.3389/fnagi.2020.616357

**Published:** 2021-01-21

**Authors:** Hidetomo Murakami, Kenjiro Ono, Tomotaka Shiraishi, Tadashi Umehara, Shusaku Omoto, Yasuyuki Iguchi

**Affiliations:** ^1^Department of Neurology, School of Medicine, The Jikei University, Tokyo, Japan; ^2^Department of Neurology, School of Medicine, Showa University, Tokyo, Japan

**Keywords:** Parkinson's disease, cerebrospinal fluid, α-synuclein, Lewy body, cognitive domain, frontal/executive function

## Abstract

The level of α-synuclein, a component of Lewy bodies, in cerebrospinal fluid (CSF) in Parkinson's disease (PD) has attracted recent attention. Most meta-analyses conclude that CSF levels of α-synuclein are decreased in PD. Patients with PD present with cognitive impairment, including frontal/executive dysfunction in the early phase and later emergence of visuospatial and mnemonic deficits. To examine whether CSF α-synuclein levels reflect the activities of various cognitive domains, we reviewed reports examining the association of these levels with cognitive performance in each domain in PD. Among 13 cross-sectional studies, five showed that a lower CSF α-synuclein level was associated with worse cognitive function. In four of these five reports, frontal/executive function showed this association, suggesting a link of the pathophysiology with Lewy bodies. In three other reports, a higher CSF α-synuclein level was associated with temporal-parieto-occipital cognitive deterioration such as memory. In the other five reports, the CSF α-synuclein level did not correlate with cognitive performance for any domain. In four longitudinal studies, a higher baseline CSF α-synuclein level was associated with a worse cognitive outcome, including cognitive processing speed, visuospatial function and memory in two, but not with any cognitive outcome in the other two. The different associations may reflect the heterogeneous pathophysiology in PD, including different pathogenic proteins, neurotransmitters. Thus, more studies of the association between cognitive domains and CSF levels of pathogenic proteins are warranted.

## Introduction

Parkinson's disease (PD) is a common neurodegenerative disease that presents with motor and various non-motor symptoms, including cognitive impairment. Such impairment of frontal/executive function, such as flexibility, planning, and working memory, is common as early cognitive impairment in PD (Williams-Gray et al., 2007; Kehagia et al., 2010). As the disease progresses, posterior cortically based cognitive deficits, such as visuospatial and mnemonic deficits, become apparent as the major symptoms of PD with dementia (PDD) (Kehagia et al., 2010; Collins and Williams-Gray, 2016). A dementia syndrome affects two or more cognitive domains and causes social and occupational impairment.

The presence of Lewy bodies consisting of α-synuclein is the pathological hallmark of the disease. According to Braak's theory, Lewy bodies are deposited in the brainstem in the early stage, then progress through the limbic and subcortical regions, and eventually are distributed over the neocortex in a later stage (Braak et al., 2003). Cerebrospinal fluid (CSF) is in close contact with the brain, and CSF levels of α-synuclein may reflect the pathology and progression of clinical symptoms. The CSF α-synuclein level has previously been compared with global/composite cognitive assessments using the mini-mental state examination (MMSE) and other scales, but no association was observed in cross-sectional (Park et al., 2011; Parnetti et al., 2011; Wennström et al., 2013) and longitudinal (Parnetti et al., 2014; Mollenhauer et al., 2019) studies. Several previous reports have compared the association of the CSF α-synuclein level with cognitive performance in each domain, but a review of the findings in these reports has not been published. Here, we review the studies that examined this association and we discuss factors that may mediate differences in correlations.

## Search Strategy and Selection Criteria

We searched for reports on correlations of CSF total α-synuclein, but not other specific types such as oligomeric and phosphorylated α-synuclein, levels and cognitive assessments in each domain in the PubMed database, using a combination of keywords of “cerebrospinal fluid, synuclein and Parkinson” with “cognitive function” or “executive” or “visuospatial” or “memory” or “attention” or “language.” Quoted papers in eligible studies were traced to ensure all relevant studies were included. Papers from any year that evaluated the relationship of the CSF α-synuclein level with assessment of a precise cognitive domain were included. The search was performed from 29th June to 1st August 2020, and 98 papers were found. However, 86 of these papers that did not mention the association between the CSF α-synuclein level and cognitive performance or that used a composite assessment such as the total MMSE or Montreal Cognitive Assessment (MoCA) score were excluded.

## Association Between the CSF α-Synuclein Level and Cognitive Performance

### Eligible Studies

Twelve papers examining associations of CSF α-synuclein levels and cognitive function in single domains were included in the study (Table 1). Three of these reports included two or three associations (Stewart et al., 2014; Compta et al., 2015; Førland et al., 2018), and 13 cross-sectional results were used from the 12 papers (Tables 1A–C). Five showed that a lower CSF α-synuclein level was associated with worse cognitive function in certain domains (Table 1A) and three showed that a higher CSF α-synuclein level was associated with worse cognitive performance in some domains (Table 1B). The other 5 results indicated that the CSF α-synuclein level did not correlate with cognitive performance in any domain (Table 1C). There were 4 longitudinal studies, of which two showed that a higher CSF α-synuclein level predicted worse cognitive outcome (Table 1D) and the other two showed no association (Tables 1E,F).

**Table 1:** Results of cross-sectional or longitudinal studies of the association between the CSF alpha-synuclein level and cognitive performance in each domain.

**Study**	**Number of participants**	**Age (range)**	**Disease duration (range)**	**H and Y (range)**	**MMSE (range)**	**Duration of follow up**	**Associated cognitive domain**	**Analysis of α-synuclein level (manufacturer of the assay)**
**A. Lower CSF** **α-synuclein indicates worse cognitive function in a crosssectional study**								
Compta et al. (2015)	PD: 41 (without/with dementia 21/20)	68/73.5^a^	10/9 years^a^	3/4^a^	28/18^a^	–	Phonetic fluency	ELISA
		(63.50–73.50)/(66.00–78.00)^b^	(7.00–15.75)/(7.25–11.50)^b^	(2–3)/(3–5)^b^	(27–29)/(16–22)^b^			(Invitrogen: KHB0061)
Compta et al. (2015)	PD without dementia: 21	68^a^	10 years^a^	3^a^	28^a^	–	Phonemic fluency,	ELISA
		(63.50–73.50)^b^	(7.00–15.75)^b^	(2–3)^b^	(27–29)^b^		visuospatial function	(Invitrogen: KHB0061)
Skogseth et al. (2015)	PD-NC and PD-MCI: 414	61.3 ± 9.7	4.0 months^a^	N.A.	N.A.	–	Phonemic fluency, attention	ELISA
		(N.A)	(2.0–8.0 months)^b^	(N.A.)	(N.A.)			(Covance)
Murakami et al. (2019)	PD, PD-MCI and PDD: 27	72.3 ± 9.2	1.3 ± 1.2 years	N.A.	21.7 ± 5.5^c^	–	Planning, executive	ELISA
		(47–84)	(0–5)	(N.A.)	(9–28)			(Covance)
Stewart et al. (2014) -phase 1	PD without dementia: 350	60.90 ± 9.21	2.08 ± 1.39 years	1.5^a^	28.86 ± 1.44	–	Memory	Luminex assay
		(34–79)	(0–7)	(1.0–2.5)	(23–30)			(Luminex)
**B. Higher CSF** **α-synuclein indicates worse cognitive function in a crosssectional study**								
Stewart et al. (2014) -phase 2	PD: 266	62.64 ± 9.03	3.80 ± 1.45 years	2.0^a^	28.74 ± 2.30	–	Verbal learning, memory	Luminex assay
		(37–80)	(1–8)	(1.0–4.0)	(8–30)			(Luminex)
Compta et al. (2015)	PD: 41(without/with dementia 21/20)	68/73.5^a^	10/9 years^a^	3/4^a^	28/18^a^	–	Naming, memory	ELISA
		(63.50–73.50)/(66.00–78.00)^b^	(7.00–15.75)/(7.25–11.50)^b^	(2–3)/(3–5)^b^	(27–29)/(16–22)^b^			(Invitrogen: KHB0061)
Wijeyekoon et al. (2020)	PD: 35	65.4 ± 7.6	5.4 ± 5.6 years	N.A.	90.3 ± 9.4^d^	–	Semantic fluency	ECL
		(N.A.)	(N.A.)	(N.A.)	(N.A.)			(Meso Scale Diagnostics)
**C. Results showing no association between CSF** **α-synuclein level and cognitive function in any domain in a crosssectional study**								
Buddhala et al. (2015)	PD without dementia: 77	66.9 ± 8.5^e^	4.9 ± 4.0 years^e^	N.A.	N.A.	–	None	ELISA
		(N.A.)	(N.A.)	(N.A.)	(N.A.)			(Covance)
Yarnall et al. (2014)	PD-NC and PD-MCI: 67	64.5 ± 9.4	5.3 ± 5.6 months	N.A.	25.2 ± 3.3^c^	–	None	ELISA
		(N.A.)	(N.A.)	(N.A.)	(N.A.)			(N.A.)
Førland et al. (2018)	PD: 56	69.1^a^	4.8 years^a^	2^a^	28.5^a^	–	None	ELISA
		(56.1–78.5)	(N.A.)	(1.0–3.0)	(23.0–30.0)			(BioLegend: #844101)
Kang et al. (2013)	PD: 63	62 ± 10	0.4 years^a^	1.65 ± 0.51	27.2 ± 2.0^c^	–	None	ELISA
		(59–64)^f^	(0.0–2.6)	(1–2)	(26.7–27.7)^f^			(Covance)
Stav et al. (2015)	PD without dementia: 31	64.65 ± 6.53	2.5 ± 1.2 years	1.5^a^	28.58 ± 1.59	–	None	ECL
		(47–74)	(1–6)	(1.0–2.5)	(22–30)			(Meso Scale Discovery)
**D. Higher CSF** **α-synuclein predicts worse outcome in cognition in a longitudinal study**								
Hall et al. (2015)	PD without dementia: 42	67.5^a^	7 years^a^	2.25^a^	29^a^	2 years	Cognitive processing speed	Luminex assay
		(60.5–73.25)^b^	(4.0–10.25)^b^	(1–2.5)^b^	(27–29)^b^			(Luminex)
Stewart et al. (2014)	PD: 266	62.64 ± 9.03	3.80 ± 1.45 years	2.0^a^	28.74 ± 2.30	Up to 6.9 years	Verbal learning, memory, visuospatial working memory	Luminex assay
		(37–80)	(1–8)	(1.0–4.0)	(8–30)	(average 1.8 years)		(Luminex)
**E. Baseline CSF** **α-synuclein level does not predict cognitive decline in a longitudinal study**								
Førland et al. (2018)	PD: 56	69.1^a^	4.8 years^a^	2^a^	28.5^a^	2 to 4 years	None	ELISA
		(56.1–78.5)	(N.A.)	(1.0–3.0)	(23.0–30.0)			(BioLegend: #844101)
**F. Change in CSF** **α-synuclein is not associated with worsening of cognitive function in a longitudinal study**								
Hall et al. (2016)	PD without dementia: 63	64.7 ± 9.4	5.5 ± 4.0 years	2.0 ± 0.7	28.5 ± 1.4	2 years	None	ELISA
		(N.A.)	(N.A.)	(N.A.)	(N.A.)			(Covance)

*Data are presented as mean (±standard deviation) unless otherwise indicated*.

a *Median*,

b *Interquartile range*,

c *Montreal cognitive assessment (MoCA)*,

d *Addenbrooke's Cognitive Examination- Revised (ACE-R)*,

e *median ± standard deviation*,

f *95% confidence interval*.

*N.A.: not available*.

*ECL, Electrochemiluminescence; ELISA, Enzyme-Linked ImmunoSorbent Assay; H and Y, Hoehn and Yahr scale; MCI, mild cognitive impairment; MMSE, Mini-mental state examination; NC, normal cognition; SD, standard deviation*.

### Cross-Sectional Assessments

#### Results Showing That a Lower CSF α-Synuclein Level Indicates Worse Frontal/Executive Cognitive Function

Four of the five reports in Table 1A showed an association of frontal/executive function with the CSF α-synuclein level. Compta et al. (2015) showed dysfunction in phonetic fluency was associated with low CSF α-synuclein in 41 PD patients with a wide range of cognitive status from normal cognition to dementia (21 patients without dementia). The CSF α-synuclein level also showed significant correlations with thickness in the frontal cortex in patients with idiopathic rapid-eye-movement sleep behavior disorder (iRBD) as a prodoromal stage of PD and PD without dementia (Compta et al., 2015). Skogseth et al. (2015) showed that low CSF α-synuclein was significantly associated with phonemic fluency and attention, but not with posterior cortical domains, such as memory and visuospatial domains, in PD and PD with mild cognitive impairment (PD-MCI). We have shown that lower CSF α-synuclein is associated with worse performance in a judgement subtest that assesses planning and executive function, but not with subtests assessing other cognitive domains in patients with PD, PD-MCI and PDD (Murakami et al., 2019). In these four studies, CSF α-synuclein levels were also correlated with CSF levels of amyloid-β 1-42 and total tau.

#### Results Showing That a Lower CSF α-Synuclein Level Indicates Worse Posterior Cognitive Function

Stewart et al. (2014) found that lower CSF α-synuclein was correlated with worse performance of memory at the beginning of phase I of the deprenyl and tocopherol antioxidative therapy of Parkinsonism (DATATOP) study, which included drug-naïve early PD patients (Table 1A).

#### Results Showing That a Higher CSF α-Synuclein Level Indicates Worse Posterior Cognitive Function

All three results in Table 1B showed an association of higher CSF α-synuclein with worse performance in posterior, but not frontal, cortical cognitive function, such as memory, naming and semantic fluency. At the beginning of phase 2 of the DATATOP study, when motor symptoms in patients on selegiline, tocopherol, both drugs or placebo had progressed to a state requiring L-dopa therapy, higher CSF α-synuclein was associated with worse verbal learning and memory score in PD without dementia (Stewart et al., 2014). Compta et al. (2015) showed that CSF α-synuclein was significantly higher in patients with a worse score for naming and memory compared to those with better scores in 41 PD patients with a wide range of cognitive function. In this cohort, CSF levels of α-synuclein correlated with those of amyloid-β 1-42 and total tau. Wijeyekoon et al. (2020) showed that the CSF α-synuclein level was negatively correlated negatively with semantic fluency score and positively with the CSF level of amyloid-β 1-42 in PD.

#### Results Showing no Association of CSF α-Synuclein Levels With Cognitive Function in any Domain

In the five results in Table 1C, the CSF α-synuclein level showed no association with any domains of cognitive assessment, including frontal/executive, attention, memory, visuospatial functions and others in PD without dementia (Buddhala et al., 2015; Stav et al., 2015), PD with normal cognition and MCI (Yarnall et al., 2014) and PD with a wide range of cognitive status (Kang et al., 2013; Førland et al., 2018). CSF α-synuclein levels were correlated with those of amyloid-β 1-42 (Buddhala et al., 2015) and total tau (Kang et al., 2013; Buddhala et al., 2015).

### Longitudinal Assessments

Two of four longitudinal studies showed that higher baseline CSF α-synuclein was associated with future worsening of cognitive function (Table 1D). Hall et al. (2015) found an association of higher baseline CSF α-synuclein with worsening in cognitive processing speed, but not with memory and word fluency at 2 years follow-up in 42 PD patients without dementia. Stewart et al. (2014) showed that lower baseline CSF α-synuclein was associated with preservation of assessment scores for verbal learning, memory and visuospatial working memory after follow-up for up to 6.9 years in PD without dementia. In contrast, Førland et al. (2018) found that CSF α-synuclein levels did not predict longitudinal cognitive decline in executive function memory and visuospatial function after 2–4 years follow-up in PD patients (Table 1E). Hall et al. (2016) followed 63 PD patients without dementia for 2 years and found that an increase in CSF α-synuclein did not correlate with a change in the letter fluency task (Table 1F).

## Possible Factors Affecting the Association Between CSF α-Synuclein Level and Cognitive Function

### Role of Change in the Dynamics of CSF α-Synuclein Over Time

Among the five cross-sectional results showing that lower CSF α-synuclein was associated with worse cognitive function, four showed an association with frontal/executive function (Table 1A). Among the cognitive domains, frontal-executive impairment is common as an early cognitive impairment in PD due to dysfunction in the frontostriatal circuit (Lewis et al., 2003; Williams-Gray et al., 2007). α-Synuclein aggregates to form oligomers that have neurotoxic effects and play an important role in the pathogenesis of PD (Ono, 2017). Meta-analyses showing that the CSF α-synuclein level in PD patients is lower than that in normal controls (Zhou et al., 2015; Eusebi et al., 2017) are consistent with aggregation and intracellular sequestering in Lewy bodies (Stewart et al., 2014; Mollenhauer et al., 2019). CSF α-synuclein in relatively early PD has been shown to decrease during 24–36 months of observation in drug-naïve PD patients (Mollenhauer et al., 2019) and during the phase I DATATOP study cohort (Stewart et al., 2014). Therefore, the association of lower CSF α-synuclein with worse frontal/executive function supports the idea that this dysfunction emerges during concurrent aggregation of α-synuclein and formation of Lewy body in relatively early PD. The finding that lower CSF α-synuclein was associated with frontal cortical thinning in patients with iRBD as a prodromal stage of PD and PD without dementia (Compta et al., 2015) supports the emergence of frontal/executive dysfunction with concurrent development of Lewy body pathology in the frontal cortex. Therefore, a lower α-synuclein level may reflect frontal/executive impairment as an early cognitive dysfunction in PD.

In contrast with the above results for frontal/executive function, there are reports showing associations of a higher CSF α-synuclein level with worse cognitive assessment in cross-sectional studies (Table 1B) and worse cognitive outcome in longitudinal studies (Table 1D). CSF α-synuclein has been found to increase over time in PD with a disease duration of >5 years (Hall et al., 2016) and in patients receiving levodopa treatment (Majbour et al., 2016). Thus, there may be a point at which CSF α-synuclein turns from decreasing to increasing in the progressive course of PD. The increase in CSF is suggested to be due to release of the pathogenic protein as a result of neural damage (Lleó et al., 2015), and these results support the idea that as neuronal damage progress, cognitive function also deteriorates. The association of high CSF α-synuclein with posterior cortical thinning in PDD also support this idea (Compta et al., 2015).

Among cognitive domains, worse posterior cortical cognitive domains, a hallmark of dementia in PD, have been associated with both higher (Table 1B) and lower (Table 1A) CSF α-synuclein levels. This opposite association may also be due to the bimodal change in CSF α-synuclein level, which decreases in the relatively early phase and increases in the later phase of PD. We found no report showing that worse frontal/executive function was associated with a higher CSF level in the search for this review. Therefore, the association of lower α-synuclein with worse frontal/executive function observed before the turning point of the CSF α-synuclein level supports frontal/executive impairment as an earlier symptom in PD.

Although we conceptually view patients in a cohort as being earlier or later stage cases with regard to the association between CSF α-synuclein and cognitive function, we cannot distinguish “earlier” or “later” patients using clinically obtainable information. For example, there are overlaps in patient background factors, such as age, disease duration and clinical symptoms, among cohort groups presenting with each type of association. However, we believe that the existence of positive and negative associations signifies differences in disease duration and pathophysiology linked to α-synuclein among the cognitive domains.

### Involvement of Pathogenic Proteins Other Than α-Synuclein

There were five results showing no association between CSF α-synuclein and cognitive assessments (Tables 1C,E,F). This indicates that the pathophysiological background of cognitive dysfunction cannot be explained by the CSF α-synuclein level as a single biomarker. There are some reports showing an association between a reduced CSF amyloid-β 1-42 level and cognitive deterioration in PD (Lim et al., 2019). Decreased CSF amyloid-β 1-42 is a confirmed biomarker for Alzheimer's disease (AD), and this decrease is suggested to be due to formation of aggregates such as senile plaque. In PD progression, posterior cortically based dysfunction is known to become dominant due to pathological findings in AD (Collins and Williams-Gray, 2016). PD often presents with an AD pathology such as senile plaques and neurofibrillary tangles (Apaydin et al., 2002), and several experimental studies have explained the co-pathology of AD and PD. α-synuclein has been shown to co-aggregate with amyloid-β 1-42 (Ono et al., 2012) and tau (Guo et al., 2013), and some of the studies in this review showed that CSF α-synuclein levels were correlated with those of amyloid-β 1-42 (Buddhala et al., 2015; Compta et al., 2015; Skogseth et al., 2015; Hall et al., 2016; Murakami et al., 2019; Wijeyekoon et al., 2020) and tau (Kang et al., 2013; Buddhala et al., 2015; Compta et al., 2015; Hall et al., 2015, 2016; Skogseth et al., 2015; Murakami et al., 2019). These reports support an interaction of α-synuclein with amyloid beta and tau in the progressive course of PD and cognitive deterioration. Amyloid-β 1-42 and tau also contribute to progression of PD, but this contribution may not be reflected by the CSF α-synuclein level.

### Involvement of Other Factors

Changes in the titers of neurotransmitters in the central nervous system during the progressive course of PD partially contribute to variation in the association between CSF α-synuclein levels and cognitive performance in each domain. Frontal/executive function in early PD is dopaminergic (Kehagia et al., 2010) and dopaminergic medication can be effective (Murakami et al., 2017). Frontal/executive function, but not memory or visuospatial function, was shown to correlate with striatal dopaminergic deficits on ^123^I-Ioflupane SPECT (Siepel et al., 2014), whereas a dopaminergic titer that is too high can impair frontal/executive function (Kehagia et al., 2010). Lewy body pathology has also been suggested to contribute to frontostriatal dopaminergic depletion and frontal atrophy (Stav et al., 2015). Therefore, the association between CSF α-synuclein level and frontal/executive function can be affected by dopamine.

Cholinergic dysfunction with an AD-like pathophysiology also contributes to cognitive deterioration in PD and has a key role in progression to PDD (Kehagia et al., 2010). Cholinergic drugs are effective for cognitive impairment in PD (Aarsland et al., 2002; Ravina et al., 2005), and cholinergic activity can affect the association between CSF α-synuclein levels and cognitive assessments. Other neurotransmitters, such as noradrenaline, also contribute to some domains of cognitive function in PD (Kehagia et al., 2010). Therefore, associations among pathological findings linked to pathogenic proteins, affected neurotransmitters, cognitive performance and lesions linked to the cognitive domain are of great interest, and it is important to determine the mechanism by which pathogenic proteins produce specificity for lesions and neurotransmitters.

PD is associated with neuronal inflammation and α-synuclein can activate microglia and astrocytes (Surendranathan et al., 2015). CSF levels of inflammatory cytokines, such as IL-1β, IL-6 and others, are increased in PD (Mogi et al., 1996; Chen et al., 2018), and the CSF α-synuclein level is associated with that of inflammation markers such as IL-1β (Hu et al., 2015), serum amyloid A (SAA), IL-8 and chitinase-3-like protein 1 (YKL-40) (Hall et al., 2018) in PD. Higher levels of CSF inflammatory markers such as CRP, SAA, IL-6 and IL-8 have been associated with worse cognition, such as cognitive speed, attention and letter fluency (Hall et al., 2018). Cortical microglial activity assessed by ^11^C-RPK11195 PET was negatively correlated with glucose metabolism in the temporo-parietal-occipital cortex, and the level of microglial activity in the temporoparietal, occipital and frontal cortex showed an inverse correlation with MMSE scores in PDD patients (Fan et al., 2015). Therefore, the status of neuronal inflammation can affect the association between the CSF α-synuclein level and cognitive function.

Various genetic factors also affect the pathology and cognitive function in PD. Correlations between cortical thinning and CSF α-synuclein levels are opposite based on two types of the SNCA rs356191 single nucleotide polymorphism, and the correlated cortical lesion also differs in PD (Sampedro et al., 2018). Some heterogeneities such as the microtubule-associated protein tau (MAPT) genotype (Williams-Gray et al., 2009) and apolipoprotein E subtype (Tsuang et al., 2013) influence development of dementia in PD. The catechol-O-methyltransferase (COMT) genotype is known to affect frontal/executive function (Williams-Gray et al., 2009). Therefore, genetic factors may also affect the association between CSF α-synuclein levels and cognitive function, and contribute to the heterogeneity in cognitive function.

In each study, CSF α-synuclein levels were assessed using different methods, such as Enzyme-Linked Immunosorbent Assay (ELISA), Electrochemiluminescence (ECL) and Luminex assay (Table 1). All 4 reports showing lower CSF α-synuclein level indicates worse frontal/executive function used ELISA (Table 1A). Whereas, studies showing the other type of association between CSF α-synuclein level and cognition used various assays (Tables 1B–F). Therefore, the method to assess CSF α-synuclein level should be paid attention in the future study.

Suggested factors that affect cognitive performance during the conceptual disease stage are summarized in Table 2.

**Table 2:** Suggested factors that affect cognitive performance during the conceptual disease stage.

Relatively early stage (Impairment in frontal/executive function is associated with lower CSF α-synuclein level)
Lewy body pathology
Dopaminergic deficit
Relatively later stage (Impairment in posterior cortically based cognitive domains are associated with higher CSF α-synuclein level)
Alzheimer's pathology
Deficit of acetylcholine or another neurotransmitter
Unspecified (unknown) stage
Neuronal inflammation
Genetic factors

## Conclusion and Future Perspective

Reports on relationships of CSF α-synuclein levels with cognitive performance in each domain have shown various associations. The type of association with the CSF α-synuclein level has tended to differ between frontal/executive and posterior cortical cognitive domains. Pathophysiology and phase of the disease in which α-synuclein pathology contributes may differ based on the cognitive domain. Therefore, it is important to focus on impairment in each cognitive domain when evaluating the pathophysiology of PD. At present, the mechanism for each type of association is uncertain, and the type of association in each cohort cannot be predicted using available information in clinical practice. Investigation of cohorts presenting with different types of association between CSF α-synuclein levels and cognitive performance in each domain will contribute to unveiling the pathophysiology of cognitive function in PD. However, as shown in this review, there are still only a few reports on associations of CSF pathogenic proteins and cognitive function. This is a limitation of the review, and further clinical studies in other cohorts and more studies on α-synuclein metabolism and PD pathogenesis are required.

## Author Contributions

HM and KO planned and wrote the manuscript. TS, TU, SO, and YI read and approved the final manuscript. All authors contributed to the article and approved the submitted version.

## Conflict of Interest

The authors declare that the research was conducted in the absence of any commercial or financial relationships that could be construed as a potential conflict of interest.

## References

[B1] Aarsland D. Laake K. Larsen J. P. Janvin C. (2002). Donepezil for cognitive impairment in Parkinson's disease: a randomised controlled study. J. Neurol. Neurosurg. Psychiatry 72, 708–712. 10.1136/jnnp.72.6.708 12023410 PMC1737925

[B2] Apaydin H. Ahlskog J. E. Parisi J. E. Boeve B. F. Dickson D. W. (2002). Parkinson disease neuropathology: later-developing dementia and loss of the levodopa response. Arch. Neurol. 59, 102–112. 10.1001/archneur.59.1.102 11790237

[B3] Braak H. Tredici K. D. Rüb U. de Vos R. A. I. Jansen Steur E. N. H. Braak E. (2003). Staging of brain pathology related to sporadic Parkinson's disease. Neurobiol. Aging 24, 197–211. 10.1016/S0197-4580(02)00065-9 12498954

[B4] Buddhala C. Campbell M. C. Perlmutter J. S. Kotzbauer P. T. (2015). Correlation between decreased CSF α-synuclein and Aβ1–42 in Parkinson disease. Neurobiol. Aging 36, 476–484. 10.1016/j.neurobiolaging.2014.07.043 25212463 PMC4268043

[B5] Chen X. Hu Y. Cao Z. Liu Q. Cheng Y. (2018). Cerebrospinal fluid inflammatory cytokine aberrations in Alzheimer's disease, Parkinson's disease and amyotrophic lateral sclerosis: A systematic review and meta-analysis. Front. Immunol. 9:2122. 10.3389/fimmu.2018.02122 30283455 PMC6156158

[B6] Collins L. M. Williams-Gray C. H. (2016). The genetic basis of cognitive impairment and dementia in Parkinson's disease. Front. Psychiatry 7:89. 10.3389/fpsyt.2016.00089 27242557 PMC4873499

[B7] Compta Y. Valente T. Saura J. Segura B. Iranzo Á. Serradell M.. (2015). Correlates of cerebrospinal fluid levels of oligomeric- and total-α-synuclein in premotor, motor and dementia stages of Parkinson's disease. J. Neurol. 262, 294–306. 10.1007/s00415-014-7560-z 25380583

[B8] Eusebi P. Giannandrea D. Biscetti L. Abraha I. Chiasserini D. Orso M.. (2017). Diagnostic utility of cerebrospinal fluid α-synuclein in Parkinson's disease: a systematic review and meta-analysis. Mov. Disord. 32, 1389–1400. 10.1002/mds.27110 28880418

[B9] Fan Z. Aman Y. Ahmed I. Chetelat G. Landeau B. Chaudhuri K. R.. (2015). Influence of microglial activation on neuronal function in Alzheimer's and Parkinson's disease dementia. Alzheimers Dement. 11, 608–621. 10.1016/j.jalz.2014.06.016 25239737

[B10] Førland M. G. Öhrfelt A. Dalen I. Tysnes O.-B. Blennow K. Zetterberg H.. (2018). Evolution of cerebrospinal fluid total α-synuclein in Parkinson's disease. Parkinson. Relat. Disord. 49, 4–8. 10.1016/j.parkreldis.2018.01.018 29409704

[B11] Guo J. L. Covell D. J. Daniels J. P. Iba M. Stieber A. Zhang B.. (2013). Distinct α-synuclein strains differentially promote tau inclusions in neurons. Cell 154, 103–117. 10.1016/j.cell.2013.05.057 23827677 PMC3820001

[B12] Hall S. Janelidze S. Surova Y. Widner H. Zetterberg H. Hansson O. (2018). Cerebrospinal fluid concentrations of inflammatory markers in Parkinson's disease and atypical parkinsonian disorders. Sci. Rep. 8:13276. 10.1038/s41598-018-31517-z 30185816 PMC6125576

[B13] Hall S. Surova Y. Öhrfelt A. Blennow K. Zetterberg H. Hansson O. (2016). Longitudinal measurements of cerebrospinal fluid biomarkers in Parkinson's disease. Mov. Disord. 31, 898–905. 10.1002/mds.26578 26878815 PMC5067556

[B14] Hall S. Surova Y. Öhrfelt A. Zetterberg H. Lindqvist D. Hansson O. (2015). CSF biomarkers and clinical progression of Parkinson disease. Neurology 84, 57–63. 10.1212/WNL.0000000000001098 25411441 PMC4336091

[B15] Hu Y. Yu S.-Y. Zuo L.-J. Cao C.-J. Wang F. Chen Z.-J.. (2015). Parkinson disease with REM sleep behavior disorder: features, α-synuclein, and inflammation. Neurology 84, 888–894. 10.1212/WNL.0000000000001308 25663225

[B16] Kang J. H. Irwin D. J. Chen-Plotkin A. S. Siderowf A. Caspell C. Coffey C. S.. (2013). Association of cerebrospinal fluid β-amyloid 1-42, T-tau, P-tau181, and α-synuclein levels with clinical features of drug-naive patients with early Parkinson disease. JAMA. Neurol. 70, 1277–1287. 10.1001/jamaneurol.2013.3861 23979011 PMC4034348

[B17] Kehagia A. A. Barker R. A. Robbins T. W. (2010). Neuropsychological and clinical heterogeneity of cognitive impairment and dementia in patients with Parkinson's disease. Lancet Neurol. 9, 1200–1213. 10.1016/S1474-4422(10)70212-X 20880750

[B18] Lewis S. J. G. Dove A. Robbins T. W. Barker R. A. Owen A. M. (2003). Cognitive impairments in early Parkinson's disease are accompanied by reductions in activity in frontostriatal neural circuitry. J. Neurosci. 23, 6351–6356. 10.1523/JNEUROSCI.23-15-06351.2003 12867520 PMC6740550

[B19] Lim E. W. Aarsland D. Ffytche D. Taddei R. N. van Wamelen D. J. Wan W. M. (2019). Amyloid-β and Parkinson's disease. J. Neurol. 266, 2605–2619. 10.1007/s00415-018-9100-8 30377818

[B20] Lleó A. Cavedo E. Parnetti L. Vanderstichele H. Herukka S. K. Andreasen N.. (2015). Cerebrospinal fluid biomarkers in trials for Alzheimer and Parkinson diseases. Nat. Rev. Neurol. 11, 41–55. 10.1038/nrneurol.2014.232 25511894

[B21] Majbour N. K. Vaikath N. N. Eusebi P. Chiasserini D. Ardah M. Varghese S.. (2016). Longitudinal changes in CSF α-synuclein species reflect Parkinson's disease progression. Mov. Disord. 31, 1535–1542. 10.1002/mds.26754 27548849

[B22] Mogi M. Harada M. Narabayashi H. Inagaki H. Minami M. Nagatsu T. (1996). Interleukin (IL)-1 beta, IL-2, IL-4, IL-6 and transforming growth factor-α levels are elevated in ventricular cerebrospinal fluid in juvenile parkinsonism and Parkinson's disease. Neurosci. Lett. 211, 13–16. 10.1016/0304-3940(96)12706-3 8809836

[B23] Mollenhauer B. Caspell-Garcia C. J. Coffey C. S. Taylor P. Singleton A. Shaw L. M.. (2019). Longitudinal analyses of cerebrospinal fluid α-Synuclein in prodromal and early Parkinson's disease. Mov. Disord. 34, 1354–1364. 10.1002/mds.27806 31361367 PMC7098385

[B24] Murakami H. Nohara T. Shozawa H. Owan Y. Kuroda T. Yano S.. (2017). Effects of dopaminergic drug adjustment on executive function in different clinical stages of Parkinson's disease. Neuropsychiatr. Dis. Treat. 13, 2719–2726. 10.2147/NDT.S145916 29123404 PMC5661838

[B25] Murakami H. Tokuda T. El-Agnaf O. M. A. Ohmichi T. Miki A. Ohashi H.. (2019). Correlated levels of cerebrospinal fluid pathogenic proteins in drug-naïve Parkinson's disease. BMC Neurol. 19:113. 10.1186/s12883-019-1346-y 31164098 PMC6549316

[B26] Ono K. (2017). The oligomer hypothesis in α-synucleinopathy. Neurochem. Res. 42, 3362–3371. 10.1007/s11064-017-2382-x 28828740

[B27] Ono K. Takahashi R. Ikeda T. Yamada M. (2012). Cross-seeding effects of amyloid β-protein and α-synuclein. J. Neurochem. 122, 883–890. 10.1111/j.1471-4159.2012.07847.x 22734715

[B28] Park M. J. Cheon S.-M. Bae H.-R. Kim S.-H. Kim J. W. (2011). Elevated levels of α-synuclein oligomer in the cerebrospinal fluid of drug-naïve patients with Parkinson's disease. J. Clin. Neurol. 7, 215–222. 10.3988/jcn.2011.7.4.215 22259618 PMC3259496

[B29] Parnetti L. Chiasserini D. Bellomo G. Giannandrea D. Carlo C. D. Qureshi M. M.. (2011). Cerebrospinal fluid Tau/α-synuclein ratio in Parkinson's disease and degenerative dementias. Mov. Disord. 26, 1428–1435. 10.1002/mds.23670 21469206

[B30] Parnetti L. Farotti L. Eusebi P. Chiasserini D. Carlo C. D. Giannandrea D.. (2014). Differential role of CSF α-synuclein species, tau, and Aβ42 in Parkinson's disease. Front. Aging Neurosci. 6:53. 10.3389/fnagi.2014.00053 24744728 PMC3978246

[B31] Ravina B. Putt M. Siderowf A. Farrar J. T. Gillespie M. Crawley A.. (2005). Donepezil for dementia in Parkinson's disease: a randomised, double blind, placebo controlled, crossover study. J. Neurol. Neurosurg. Psychiatry 76, 934–939. 10.1136/jnnp.2004.050682 15965198 PMC1739697

[B32] Sampedro F. Marín-Lahoz J. Martínez-Horta S. Pagonabarraga J. Kulisevsky J. (2018). Cortical thinning associated with age and CSF biomarkers in early Parkinson's disease is modified by the SNCA rs356181 polymorphism. Neurodegener. Dis. 18, 233–238. 10.1159/000493103 30336481

[B33] Siepel F. J. Brønnick K. S. Booij J. Ravina B. M. Lebedev A. V. Pereira J. B.. (2014). Cognitive executive impairment and dopaminergic deficits in de novo Parkinson's disease. Mov. Disord. 29, 1802–1808. 10.1002/mds.26051 25284687

[B34] Skogseth R. E. Bronnick K. Pereira J. B. Mollenhauer B. Weintraub D. Fladby T.. (2015). Associations between cerebrospinal fluid biomarkers and cognition in early untreated Parkinson's disease. J. Parkinson. Dis. 5, 783–792. 10.3233/JPD-150682 26599300 PMC5031486

[B35] Stav A. L. Aarsland D. Johansen K. K. Hessen E. Auning E. Fladby T. (2015). Amyloid-β and α-synuclein cerebrospinal fluid biomarkers and cognition in early Parkinson's disease. Parkinson. Relat. Disord. 21, 758–764. 10.1016/j.parkreldis.2015.04.027 25971633

[B36] Stewart T. Liu C. Ginghina C. Cain K. C. Auinger P. Cholerton B.. (2014). Cerebrospinal fluid α-synuclein predicts cognitive decline in Parkinson disease progression in the DATATOP cohort. Am. J. Pathol. 184, 966–975. 10.1016/j.ajpath.2013.12.007 24625392 PMC3969999

[B37] Surendranathan A. Rowe J. B. O'Brien J. T. (2015). Neuroinflammation in Lewy body dementia. Parkinson. Relat. Disord. 21, 1398–1406. 10.1016/j.parkreldis.2015.10.009 26493111

[B38] Tsuang D. Leverenz J. B. Lopez O. L. Hamilton R. L. Bennett D. A. Schneider J. A.. (2013). APOE ε4 increases risk for dementia in pure synucleinopathies. JAMA. Neurol. 70, 223–228. 10.1001/jamaneurol.2013.600 23407718 PMC3580799

[B39] Wennström M. Surova Y. Hall S. Nilsson C. Minthon L. Boström F.. (2013). Low CSF levels of both α-synuclein and the α-synuclein cleaving enzyme neurosin in patients with synucleinopathy. PLoS ONE 8:e53250. 10.1371/journal.pone.0053250 23308173 PMC3540093

[B40] Wijeyekoon R. S. Moore S. F. Farrell K. Breen D. P. Barker R. A. Williams-Gray C. H. (2020). Cerebrospinal fluid cytokines and neurodegeneration-associated proteins in Parkinson's disease. Mov. Disord. 35, 1062–1066. 10.1002/mds.28015 32413185 PMC8629119

[B41] Williams-Gray C. H. Evans J. R. Goris A. Foltynie T. Ban M. Robbins T. W.. (2009). The distinct cognitive syndromes of Parkinson's disease: 5 year follow-up of the CamPaIGN cohort. Brain 132, 2958–2969. 10.1093/brain/awp245 19812213

[B42] Williams-Gray C. H Foltynie T. Brayne C. E. G. Robbins T. W. Barker R. A. (2007). Evolution of cognitive dysfunction in an incident Parkinson's disease cohort. Brain 130, 1787–1798. 10.1093/brain/awm111 17535834

[B43] Yarnall A. J. Breen D. P. Duncan G. W. Khoo T. K. Coleman S. Y. Firbank M. J.. (2014). Characterizing mild cognitive impairment in incident Parkinson disease: the ICICLE-PD study. Neurology 82, 308–316. 10.1212/WNL.0000000000000066 24363137 PMC3929202

[B44] Zhou B. Wen M. Yu W.-F. Zhang C.-L. Jiao L. (2015). The diagnostic and differential diagnosis utility of cerebrospinal fluid α -Synuclein levels in Parkinson's disease: a meta-analysis. Parkinson. Dis. 2015:567386. 10.1155/2015/567386 26336612 PMC4532865

